# Continuous Renal Replacement Therapy for Two Neonates With Hyperammonemia

**DOI:** 10.3389/fped.2021.732354

**Published:** 2021-11-05

**Authors:** Christopher Markham, Caroline Williams, Cory Miller, Dorothy K. Grange, T. Keefe Davis, Kenneth E. Remy

**Affiliations:** ^1^Department of Pediatrics, Washington University School of Medicine, St. Louis, MO, United States; ^2^Department of Internal Medicine, Washington University School of Medicine, St. Louis, MO, United States

**Keywords:** hyperammonemia, hemodialysis, neonate, citrullinemia, methylmalonic acidemia

## Abstract

**Objectives:** This study aims to assess the feasibility of using hemofiltration for ammonia clearance in low body weight infants with an inborn error of metabolism.

**Design:** A study of two cases.

**Setting:** Quaternary pediatric hospital (Saint Louis Children's Hospital) NICU and PICU.

**Patients:** Infants <6 months of age with an ICD-9 diagnosis of 270.6 (hyperammonemia).

**Interventions:** Continuous renal replacement therapy (CRRT).

**Measurements and Main Results:** We measure serum ammonia levels over time and the rate of ammonia clearance over time. Continuous renal replacement therapy was more effective than scavenger therapy alone (Ammonul™) for rapid removal of ammonia in low weight infants (as low as 2.5 kg).

**Conclusions:** Continuous renal replacement therapy is technically feasible in low weight infants with severe hyperammonemia secondary to an inborn error of metabolism.

## Introduction

Pediatric inborn errors of metabolism that cause hyperammonemia pose specific technical challenges in critical care management, especially in the newborn period when presenting in extremis. During this newborn period, both urea cycle defects (UCDs) or organic acidemias commonly present with hyperammonemia. Additionally, to a lesser extent, some patients with maple syrup urine disease and fatty acid oxidation disorders can also have mildly elevated ammonia. Infants with these aforementioned diseases commonly present with decreased levels of consciousness and poor feeding, while up to 50% can also present with seizures (1). Appropriate and prompt therapy is paramount to improved morbidity and mortality outcomes.

The ammonia scavenging agent sodium acetate and sodium phenylbutyrate (Ammonul™) has been successful in improving outcomes for this patient population. Previous reports by Enns et al. show improved survival of up to 84% in children with hyperammonemia—a staggering improvement when directly compared to the 84% mortality that Nasogne et al. reported during the same time period for patients that did not receive sodium acetate and sodium phenylbutyrate (2, 3). Despite the improved outcomes achieved with ammonia scavenging therapy, mortality for neonates with severely elevated serum ammonia remains high. Subgroup analysis of the Enns cohort shows that neonates with ammonia levels >1,000 μmol/L (1,804 μg/dl) at presentation have a survival rate of <50% (3).

For neonates with severe UCDs or other in-born errors of metabolism, continuous renal replacement therapies (CRRT) including hemofiltration may be beneficial. Unfortunately, the use of hemodialysis for newborns with hyperammonemia is technically difficult due to vascular access and the extracorporeal circuit volume which is large in proportion to an infant's circulating blood volume of 75–90 ml/kg. Additionally, RRT requires high blood flow and dialysate rates (in ml/min) that have the potential to cause circulatory collapse and/or cardiac arrest. Hemodialysis alone is often associated with rebound hyperammonemia (4). Consequently, practitioners have considered other modes of dialysis and found that using CRRT with increased blood flow and dialysate flow can be effective (4, 5).

With careful considerations given to obtaining vascular access and maintaining adequate hemodynamic support, renal replacement therapy can be effective even for low birth weight infants. Our aim with this study is to assess the feasibility of CRRT in infants presenting with severe hyperammonemia. We hypothesize that dialysis is an effective method for ammonia clearance in this patient population and careful consideration for its deployment may impact overall outcomes.

## Methods

We conducted a retrospective cohort study in children admitted from 2009 to 2015 presenting with emergencies from inborn errors of metabolism at a single center, quaternary pediatric hospital. Local IRB approved the study design and waived the need to obtain informed consent (Washington University IRB# 201506089). Patients were enrolled with the following inclusion criteria: hospitalized infants <6 months of age AND a diagnosis of hyperammonemia secondary to an inborn error of metabolism, AND who received CRRT for ammonia reduction. We excluded any patients who received prior HD or who had CRRT for reasons other than ammonia clearance.

The initial database screen was performed by one study investigator who queried the hospital's billing database for an ICD-9 code of 270.6 (hyperammonemia). This initial screen was further narrowed by two separate investigators who identified patients that met enrollment criteria for the study. Descriptive data is presented in each case as available in the electronic medical record.

## Results

From 2009 to 2015, there were 138 inpatients with an ICD-9 coding diagnosis of hyperammonemia. Of those 138 patients, six received CRRT during their hospital course and two met inclusion criteria. All patient exclusions occurred as patients were >6 months of age. Clinical data include demographic and relevant laboratory data are summarized in Table 1.

**Table 1:** Patient demographics and initial laboratory values.

	**Patient A**	**Patient B**
Age	4 days	3 days
Gender	F	M
Weight (kg)	2.52	2.92
Body surface area	0.19	0.2
Lactate	4.7	NA
Sodium	154	151
Potassium	4.9	4.4
Chloride	125	118
Bicarbonate (CO_2_)	15	<5
Urea nitrogen	9	29
AST	61	241
ALT	24	41
Creatinine	1.5	0.7
Total calcium	6.3	7.1

### Case A

#### Presentation

A 4 day old, Indian-American female that initially presented to an outside emergency department for poor feeding and grunting. Upon arrival to the ED, the patient was “sluggish,” hypothermic to 35.5°C, and noted to have hypoxemia with SpO_2_ <90%. A point of care (POC) glucose was <1.11 mmol/L. The patient was given a bolus of D10 and D5 normal saline (NS) with a repeat blood glucose value 1 h later of 3.5 mmol/L. Blood cultures, complete blood count (CBC), and electrolyte panel were collected with a significant finding of a white blood cell (WBC) of 19.1 k/cumm. En route to the ED, the patient required bag mask positive pressure ventilation for shallow respirations and persistent hypoxemia. With signs of poor perfusion, posturing, and apnea the patient was promptly intubated. After intubation, she was hypotensive (30/20), requiring an additional 20 ml/kg NS fluid bolus. Additionally, she was given phenobarbital (20 mg/kg) for rhythmic tonic-clonic movements concerning for seizures. Given findings of hypoxemia, hypotension, new seizure disorder, hypoglycemia, and hypothermia, neonatal sepsis was considered with ampicillin and cefotaxime antibiotics and acyclovir given in the ED. The patient was promptly transported to the pediatric intensive care unit (PICU) and noted to have significant ammonia and lactate (1,382 μg/dl and 4.4 mmol/L, respectively) levels. Other notable labs include aspartate transaminase (AST) of 61 units/L and alanine transaminase (ALT) of 24 units/L. Given a concern for an inborn error of metabolism, general surgery was consulted and placed an emergent dialysis catheter at the bedside. A 7 French apheresis catheter was placed in the right internal jugular vein with a venous cut down approach. Prior to catheter placement, vital signs were significant for: HR 209, RR 40 (intubated, sedated, on neuromuscular blockade), T 36.1°C, BP 94/67, SpO_2_ 94% on 2 epinephrine infusion (up to 0.1 mcg/kg/min) and given calcium gluconate boluses of 100 mg/kg secondary to hemodynamic instability after central line placement but prior to CRRT initiation.

#### Technical Considerations

**Figure 1:**
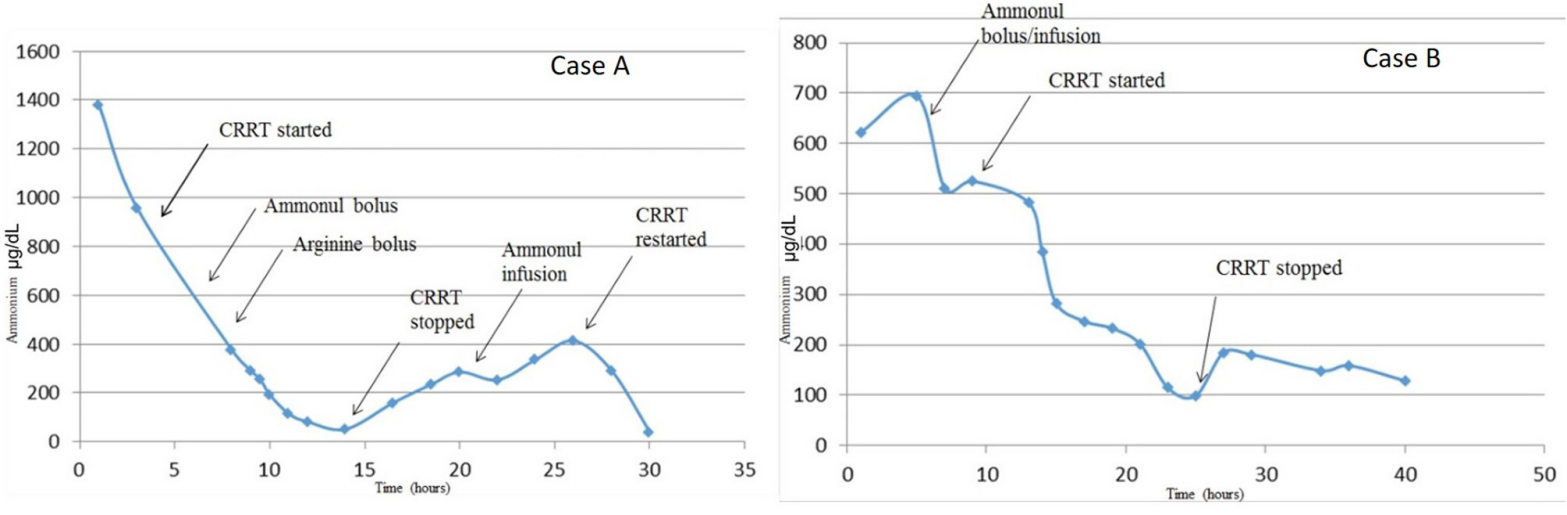
Serum ammonia levels from time of admission to resolution of hyperammonemia. Serum ammonia levels substantially decreased with the addition of CRRT to pharmacologic scavenger therapies (Ammonul™). Although both therapies were effective in decreasing serum ammonia levels, it appears that CRRT combined with Ammonul™ provides a more robust decrease. CRRT rate settings in both cases were: blood flow of 40 ml/min, dialysate flow of 1,400 ml/h, and replacement flow of 200 ml/h.

For CRRT initiation, the circuit was primed with cross-matched blood mixed with NS and 5,000 units/L heparin to hematocrit of 30%. High dose CRRT was prescribed, targeting dialysate and replacement fluid flow rates of 8,000 ml/h/1.73 m^2^ vs. conventional dosing of only 2,000 ml/h/1.73 m^2^, as recommended for infants with hyperammonemia (6). The CRRT was initiated using Prismasol™ solution containing 4 mEq/L of potassium and 32 mEq/L bicarbonate. At initiation of CRRT, the patient did not have any worsening of hemodynamic instability that required either escalation of inotropic medications (i.e., epinephrine) s or addition of alternative inotropes or vasopressors. Vital signs at the time of initiation were: T35.5°C, HR 148, RR 42, BP 94/67, 96% on FiO_2_ of 0.6. She tolerated a blood flow rate of 40 ml/min, a dialysate flow rate of 1,400 ml/h, and a replacement fluid rate of 200 ml/h. Anticoagulation was maintained with citrate dextrose solution at 30 ml/h titrated for a goal circuit ionized calcium of <0.5 mmol/L, which provided an anticoagulation effect of aPTT 34–45 s (1.5–2.0 X normal). Electrolytes were monitored at a minimum of every 4 h during high dose CRRT due to risk of electrolyte imbalance. The patient did not have any hypokalemia or worsening of hypothermia during her circuit run; asymptomatic hypophosphatemia did occur and was corrected with intravenous potassium phosphate infusions. After 10 h of CRRT, her ammonia level was <60 μg/dl and CRRT was discontinued. Figure 1 demonstrates serial serum ammonia levels and rate of ammonia clearance. Twelve hours after the CRRT was discontinued the patient had a significant rebound of serum ammonia level which required re-initiation of CRRT for an additional 4 h. Scavenger therapy was maintained during the peri-CRRT period for theoretical additive effect.

#### Patient Outcome

Due to significant disease related neurologic dysfunction over the 24 h post CRRT initiation, the family decided to redirect her goals of care to comfort measures and the patient died the next day. Her diagnostic postmortem work-up revealed a diagnosis of citrullinemia.

### Case B

#### Presentation

A 3 day old Caucasian male that presented to an outside hospital and was noted to be “listless with poor feeding and tone.” In initial assessment he was noted to be hypothermic at 32.5°C with delayed capillary refill and a POC glucose of 1.89 mmol/L. He was given 6 ml of d10 water with improvement in glucose to 3.39 mmol/L. He was fluid resuscitated for hypotension with 50 ml of NS for a systolic blood pressure of 68 mmHg. Blood, urine, and cerebrospinal fluid cultures were collected and he was started on empiric antibiotics with ampicillin and gentamicin prior to ICU transfer. Upon arrival to the ICU, his temperature was 36.7°C with pulse 136, respiratory rate 60, and blood pressure of 71/26 via non-invasive automated sphygmomanometer. His physical examination included a capillary refill <2 s, clear to auscultation lung fields, no presence of cardiac murmurs, and a liver palpated 2 cm below the right costal margin. He demonstrated minimal response to stimulation and did not exhibit a suck reflex. An umbilical arterial catheter and double lumen umbilical venous catheter were placed shortly after admission and 10% dextrose containing fluids were started at a GIR of 7 mg/kg/min. Initial laboratory screens included an ammonia of 623 μg/dl, ALT of 41 units/L, AST of 241 units/L, and an arterial blood gas with pH of 7.06, PaCO_2_ of 13 mmHg, PaO_2_ of 151 mmHg, and base deficit of 26.5 mEq/L. Surgery was consulted for vascular dialysis catheter placement in the operating room; a 7 French apheresis catheter was placed in his right internal jugular vein using a venous cut down approach without complications. Prior to hemodialysis catheter placement, he received Ammonul loading dose of 720 mg (246 mg/kg) followed by a continuous infusion of 10.4 mg/kg/h.

#### Technical Considerations

**Figure 2:**
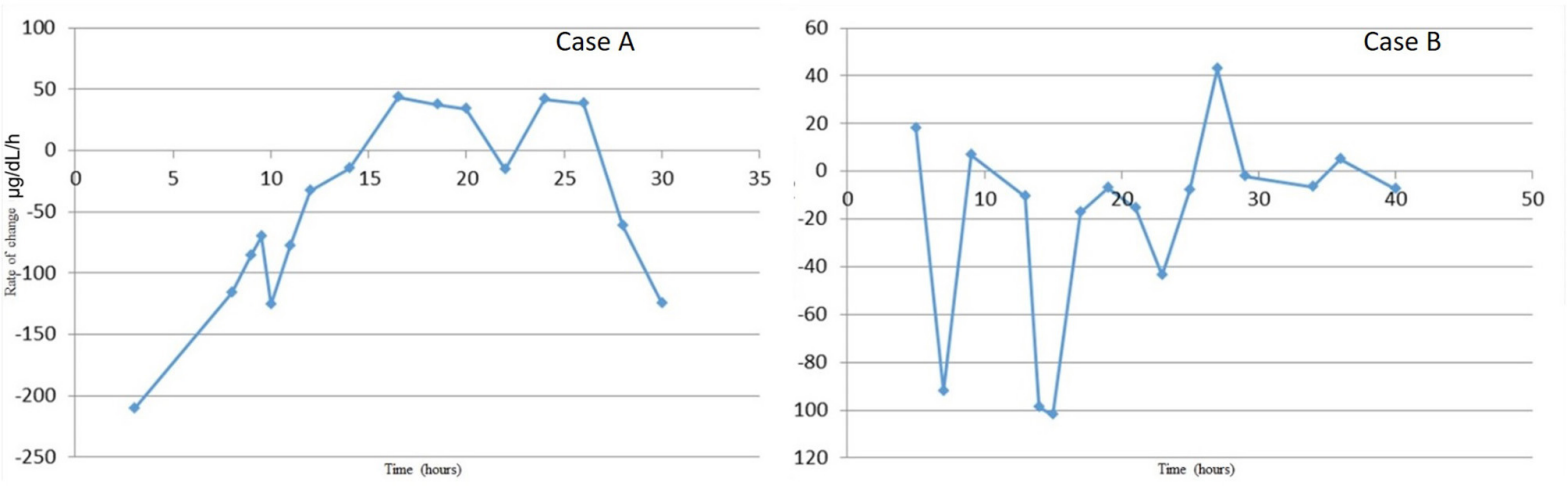
Rate of ammonia clearance from time of admission to resolution of hyperammonemia. This was determined as the slope between each proximal pair of serum ammonia measurements. The rate of change is most significant with initial fluid resuscitation and then again with combination therapy of ammonium scavengers and CRRT.

The CRRT circuit was primed with cross-matched blood mixed with NS and 5,000 units/L heparin to hematocrit of 30%. As previously recommended, high dose CRRT was prescribed, targeting dialysate, and replacement fluid flow rates of 8,000 ml/h/1.73m^2^ (6). The patient's vitals at circuit initiation were 36.9°C, HR 129, RR 32 (intubated, sedated, and on neuromuscular blockade after the OR course), and BP 66/36. With hemodynamic instability, he was given a bolus of calcium gluconate 100 mg/kg. After initiation of CRRT using Prismasol™ solution containing 4 and 32 mEq/L bicarbonate, the patient tolerated a blood flow rate of 40 ml/min, a dialysate flow rate of 1,400 ml/h, and a replacement flow rate of 200 ml/h. Anticoagulation was maintained with citrate dextrose solution at 56 ml/h titrated for goal circuit ionized calcium of < 0.5 mmol/L. Continuous renal replacement therapy was discontinued after 15 h once goal ammonia levels (<100 μg/dl) were achieved. During his circuit run, he did not have any hypokalemia or hypothermia. Figure 2 summarizes the patient's serial serum ammonia levels and rate of ammonia clearance.

#### Patient Outcome

The patient was discharged from the hospital with a new diagnosis of methylmalonic acidemia.

## Discussion

Hyperammonemia is a clinical finding with life-threatening and life-altering implications. If left untreated, hyperammonemia can lead to irreversible brain injury especially in the developing brain. This injury may result in intractable seizures, motor and/or cognitive impairment, cerebral palsy, and even death (7). It is hypothesized that hyperammonemia causes irreversible brain damage causally through its glutamine production metabolic pathway. More specifically, in astrocytes ammonia is converted into glutamine via the glutamine synthase enzyme. In physiologic environments, this enzymatic pathway exists at near capacity, making it easily saturated under increased ammonia production. Glutamine is transported to extracellular fluid via the SNAT5 transporters and is eventually taken up by neighboring neurons where is enzymatically converted to glutamate via glutaminase. Glutamate is then released into the synaptic cleft where it activates NDMA receptors which leads to excito-toxicity. This NMDA-receptor activation further potentiates increased intracranial pressure through the activation of nitric-oxide synthetase which synthesizes nitric oxide, a vasodilator (8). Additionally, glutamate is an osmotic agent and is hypothesized to contribute to the cerebral edema seen in patients with hyperammonemia. Furthermore, glutamine in mitochondria is converted back into ammonia through the phosphate-activated glutaminase. Ammonia is known to increase mitochondrial permeability leading to mitochondrial swelling and dysfunction, ultimately leading to cellular death (9).

A thorough understanding of the pathophysiology of hyperammonemia is crucial in improving the outcomes associated with the hyperammonemia secondary to UCDs and mitigating its consequences. In Japan, Uchino et al. investigated the clinical outcome and prognosis of 216 patient diagnosed with UCDs between 1978 and 1995. By1995, the 5-year survival rate was 22% for neonatal-onset and 41% for late-onset UCDs. Among the 20 long-term survivors of the neonatal-onset UCDs, 90% (18/20) had moderate-to-severe neurodevelopmental deficits compared to 28% (13/47) of patients with late-onset development of UCDs. When analyzing 108 of these patients, peak ammonia level during the first hyperammonemic attack was correlated to neurodevelopmental outcome. Specifically, when peak ammonia levels were <306 μg/dl (five times upper limit of normal), there were no severe neurological deficits; however, when peak ammonia levels were >596 μg/dl (10 times upper limit of normal), patients either died or had severe neurological deficits (10). The hypothesis that peak ammonia levels may have a significant correlation with clinical outcome is also supported by Claude Bachmann who performed a retrospective evaluation on 88 patients with UCDs and found that when peak ammonia levels were ≥817 μg/dl, none of the patients had a normal neurological outcome (11). In contrast, Msall et al., found in 26 patients with neonatal UCDs that there was no significant correlation between peak ammonia levels and IQ, but rather a significant negative linear correlation (*r* = −0.72, *p* < 0.001) between the duration of a stage III or IV hyperammonemic coma and IQ at 12 months of age (12). Though the clinical outcomes of neonates with hyperammonemia secondary to UCDs would benefit from larger retrospective analyses, the initial findings of these studies all highlight the importance of timely diagnoses and prompt reduction of ammonia levels.

In our case series, we describe two patients with similar presentations of severe hyperammonemia, due to different metabolic diseases, and differing outcomes. There are a number of similarities and key differences in disease presentations between both newborns. Both patients presented as small for gestational age, within the first week of life, and with severe dehydration. More specifically, Case A presented a day later than Case B, which may explain the severity of her clinical course including worsened initial acidosis and presenting ammonia level more than twice that of Case B. Despite these differences, both patients achieve rapid ammonia clearance. Figures 1, 2 both show that when high dose hemo-dialysis-filtration is applied to standard ammonia scavenging therapies, serum ammonia can rapidly decline to normal levels in <24 h.

There are some additional differences between the presented two cases including the use of Arginine administration in Case A as compared to the decision to forego this therapy in Case B. First, although neither patient had a diagnosis prior to medical scavenger therapies, arginine therapy is not without hypothetical risk i.e., if patient A had arginase deficiency, then arginine would be contraindicated. However, given the unlikely diagnosis of arginase deficiency with a severe clinical presentation, particularly with such a high ammonia level, incongruence with typical presentation later in life, and without such profoundly high levels of ammonia, arginine was given in patient A.

Additionally, both patients experience a rebound in ammonia levels just discontinuation of CRRT. We postulate that the scavenger therapies were not yet at steady state, potentially due to the clearance of this therapy because of high dose CRRT and the patients remained in a catabolic state causing an increase in ammonia. Furthermore, aggressive alimentation was not pursued during the period of CRRT; while both infants were started on appropriate initial GIR fluids, neither were escalated on dextrose containing fluids, started on lipid infusions, nor insulin infusions, all of which are methods to prevent further catabolism. The rebound phenomenon our patients experienced may be in part because of the kidney replacement strategy. Ammonia has a high volume of distribution, and therefore redistribution from extravascular space occurs. As this is a recognized and expected occurrence, some centers report using high-flux CRRT until ammonia levels return to goal range, and then continuing regular flow CRRT for another 12–24 h (13, 14). This case series adds support to that strategy.

Figure 1 reinforces some key clinical pearls in the treatment of hyperammonemia. Both patients have an initial improvement in serum ammonia levels secondary to stabilization and rehydration with IV fluids. There are several possible contributing factors that may explain why Case A has a more significant rebound of ammonia than Case B. One of these factors is a delay in maintenance Ammonul initiation for Case A as a result of a pharmaceutical error; this error underscores the efficacy of ammonia scavengers in serum ammonia reduction. However, in severe enzyme deficiencies and in cases of severe hyperammonemia, medical therapy alone is often not sufficiently quick or robust. Another possible confounder in the rate of rebound between the two patients is their separate enzyme deficiencies. Given their differing diagnoses, a direct comparison of rebound hyperammonemia becomes difficult to interpret.

Other case reports and series have also utilized CRRT, though strategies have differed temporally to ammonia reduction. For instance although largely different from our strategy, Kim et al. utilized CRRT to reduce ammonia levels in a newborn with ornithine transcarbamylase deficiency (OTC), a common UCD, though they continued CRRT for 7 days while using blood flow rates of 25 ml/min, with a replacement and dialysate rate both at 150 ml/h. Additionally, this group did not utilize a scavenger therapy. Using their strategy, the patient's ammonia was reduced from a peak of >1, to 422 μg/dl at 24 h, and 222 μg/dl at 57 h. The patient had a rebound in ammonia 364 μg/dl after 5 days of dialysis and therefore continued on CRRT until 7 days (15). Another case series by Santa Maria et al. included six newborns with UCDs who were placed on CRRT. Of these six, four were placed on hemo-dialysis-filtration. The average length of time on dialysis for all six patients was 49.5 h, a noticeable difference to the timing required by the two patients in our study. In regards to outcomes, two of the six patients died and only one of the four survivors had neurological deficits at follow-up 2.3 years later (16). Furthermore, our approach to CRRT therapy aligns with Spinale et al. who presented two case reports of infants with OTC presenting within 1 week of life with hyperammonemia (peak ammonia levels of 2,475 and 2,362 μg/dl, respectively) where the treatment team utilized CRRT with dialysate and replacement fluid rates of 8,000 ml/h/1.73 m^2^ (four times higher than normal dialysate/replacement fluid rates used in acute kidney injuries). They found that the ammonia for both infants decreased to <681 μg/dl within 2 h, and <170 μg/dl within 10 h. Both infants had mild rebound in ammonia levels to 254 and 283 μg/dl (with the second infant having <170 μg/dl after 24 h of discontinuation of CRRT) (6).

The use of CRRT therapy in <3 kg infants has some important technical considerations. Care must be taken when initiating CRRT for small infants as the circuit volume is a significant percentage of their circulating blood volume. The extracorporeal circuit used for both patients in this series was 72 ml. If an estimated blood volume for this age range is 80 ml/kg, with Patient A weighing 2.52 kg (body surface area of 0.19 m^2^) and Patient B weighing 2.92 kg (body surface area of 0.20 m^2^), the extracorporeal volume is 36 and 31%, respectively. Preparation and prevention for impending hemodynamic instability when initiating CRRT is necessary with the consideration and use of inotropes in addition to priming the circuit with diluted RBCs.

Additionally, close monitoring for hypocalcemia is required. High dialysate prescription rates based upon effluent dose can result in total calcium depletion if calcium is not used in dialysate replacement fluids—which was the case at the time these patients received renal replacement therapy. Furthermore, this patient population often presents in extremis with severe acidosis. As this acidosis is corrected by care teams, these infants have a calcium shift from extracellular space to intracellular space. Finally, there is a high incidence of citrate toxicity in patients with inborn errors of metabolism as a result of poor hepatic function (both from hypo-perfusion as well as immaturity) which further compounds the risk of hypocalcemia. Although we believe the risk of citrate toxicity was low in these patients because citrate is based upon blood flow (and not effluent rate) as well as allowing for higher post filter ionized calcium levels, the risk of hypocalcemia is high for the multitude of reasons detailed here. Calcium and electrolyte levels need to be monitored frequently, and both patients in this series needed boluses of calcium gluconate on top of the calcium infusions given for reversal of citrate anticoagulation.

Our case series has some limitations. Although 138 patients diagnosed with hyperammonemia were screened for this study, only two met inclusion criteria. The most likely explanation for this is sampling bias from the retrospective nature of the study. As a result, we are only able to make definitive conclusion on the logistics of clearing ammonia in low birth weight infants via CRRT as opposed to drawing conclusions on therapeutic efficacy. For instance, despite adequate ammonia clearance, Patient A did not have meaningful neurologic recovery, which we attribute to both the severity of her enzyme deficiency and her significant disease process at the time of presentation. In comparison, the use of ammonia scavenging therapies in conjunction with the use of CRRT rapidly decreased the ammonia levels in Patient B who subsequently survived his hospitalization. This difference in patient outcome is likely secondary to differences in enzyme deficiency and severity of illness at presentation.

Nonetheless, in this case-series, we show that CRRT is technically feasible in low weight infants with severe hyperammonemia secondary to inborn errors of metabolism. Undeniably barriers to ammonia clearance are logistical and primarily involve technical challenges associated with patient access and hemodynamic instability—placement of a large enough catheter to perform dialysis at high blood flow rates as well as careful monitoring of hemodynamics given the high ratio of extracorporeal volume to patient blood volume. Further studies emphasizing earlier diagnosis and rapid deployment of CRRT across centers will likely yield the greatest improvements in morbidity and mortality for this patient population. Additionally, future studies evaluating rapid CRRT initiation for severe hyperammonemia secondary to inborn errors of metabolism are needed to help clarify aspects of care teasing out timing of initiation, cases that might be ammendale to scavenger therapy alone, and prognostic factors of worsened outcomes for this patient population.

## Author Contributions

CM conceptualized and designed the study, carried out the initial analyses, drafted the initial manuscript, and approved the final manuscript as submitted. CW designed the data collection instruments, coordinated and supervised data collection, critically reviewed the manuscript, and approved the final manuscript as submitted. DG, TD, and CM assisted in study design, critically reviewed the manuscript, and approved the final manuscript as submitted. KR conceptualized and designed the study, reviewed and revised the manuscript, and approved the final manuscript as submitted. All authors contributed to the article and approved the submitted version.

## Funding

KR is supported by the National Institutes of Health NIGMS 5K08GM129763.

## Conflict of Interest

The authors declare that the research was conducted in the absence of any commercial or financial relationships that could be construed as a potential conflict of interest.

## Publisher's Note

All claims expressed in this article are solely those of the authors and do not necessarily represent those of their affiliated organizations, or those of the publisher, the editors and the reviewers. Any product that may be evaluated in this article, or claim that may be made by its manufacturer, is not guaranteed or endorsed by the publisher.
